# Mind the Gap: From Tool to Knowledge Base

**DOI:** 10.1089/bio.2018.0018

**Published:** 2018-12-17

**Authors:** Michaela Th. Mayrhofer, Irene Schlünder

**Affiliations:** ^1^ BBMRI-ERIC, Graz, Austria.; ^2^ TMF, Berlin, Germany.

**Keywords:** knowledge base, ELSI, service, guidance, data sharing, data protection

## Abstract

With the ethical, legal, and societal issues (ELSI) Knowledge Base, we introduce a key element of the Biobanking and Biomolecular Resources Research Infrastructure—European Research Infrastructure Consortium (BBMRI-ERIC) Common Service ELSI, which provides ethical, legal, and societal support for researchers and biobankers involved in transnational research. In contrast to the customized support provided by the ELSI Helpdesk, the ELSI Knowledge Base will be available to the user on a self-serve basis. The information that is made available through a knowledge base comes from multiple sources, usually from several expert contributors who are well versed in the subject matter. The knowledge base provides users with a first orientation on the subject matter, as well as allowing them to explore more detailed information if desired in a self-service manner. It is crucial that the information and knowledge provided are shared in a manner that is user friendly. Long lists of links, legalistic language, and multiple links have to be avoided wherever possible. The long-term sustainability and accuracy of a knowledge base need to be ensured by placing its expert curation and technical maintenance under the responsibility of an organization rather than a research consortium. In its core, it builds on a scenario-based approach using a nonlegalistic language. In addition, the knowledge base connects to frequently asked questions, promotes contract and informed consent templates, how-to-guides, best-practice models, and scripts. The ELSI Knowledge Base is a key element of the BBMRI-ERIC Common Service ELSI, which currently serves biobanks but will be enlarged to serve the biological and medical sciences community. In contrast to the ELSI Helpdesk, which provides customized support, the ELSI Knowledge Base is available to the user on a self-serve basis. The conceptualization of the ELSI Knowledge Base builds on assessments of several ethical, legal, and societal guidance tools that favor a single sustainable knowledge base for closing the knowledge gap by providing practical hands-on guidance for researchers. Ultimately, the ELSI Knowledge Base aims at promoting practical know-how and skills for conducting responsible research.

## Introduction

This article concentrates on ethical and legal guidance for researchers in practice and presents a novel resource for guiding researchers and promoting knowledge in ethical, legal, and societal matters in the form of a knowledge base.

However, why is a novel approach necessary and what should it achieve? Identifying and navigating through the applicable ethical and legal requirements for conducting biomedical research can be quite a practical challenge for investigators, especially when a research project encompasses participants from more than two countries and jurisdictions. To use biological material and data of human origin in a transnational research context, it has to be taken into account that issues that are well-regulated in one country by a single law (e.g., Biobank Act in Finland^1,2^), might be regulated by a set of laws in another (e.g., France^3^). As a result, practical complications typically follow suit that were either unknown or underestimated when conceiving the project scientifically.^*^

Even though there is a strong consensus with regard to basic ethical requirements, respective procedures and incentives for adherence to them might differ locally and are often enforced indirectly by the funder's requirements. In case a research project is supported by the European Union (EU), the application of fundamental ethical principles and legislation is enforced through the EU's Ethics Appraisal Procedure.^4^ This procedure refers to the EU's process to assess and address the ethical dimension of activities funded under Horizon 2020 in all possible domains of research, from the conceptual stage of the proposal to the conducted research. In other words, the EU requires the investigators to consider and show ethical adherence as a requirement to receive a research grant. This makes the EU an important and active agent in the promotion of influential soft law, such as the UNESCO declarations^5^ that specifically address human rights in biomedical research and genetics, and the Helsinki Declaration^6^ that sets forth principles on medical research involving human subjects, or the Council of Europe's^7^ adopted recommendations on research on biological materials of human origins, especially in relation to removal, storage, and use. While global and European soft law instruments set both common and universal principles, they leave considerable discretion to national jurisdictions in how these principles have to be followed. National discretion is used in predominantly two ways: Member States have the possibility to (a) accede to the relevant international treaties that regulate a specific subject matter or (b) regulate these questions domestically (or not). Even when adhering to international treaties, different countries might implement certain obligations differently.

Research, of course, is a largely transnational endeavor. Samples and/or associated data from patients or research participants are shared and used across international research teams. Research data are stored in large data sets and the secondary use of data is promoted. Moreover, research data are increasingly expected to follow the FAIR Principles (Findability, Accessibility, Interoperability, and Reusability)^8^ to improve data availability and reusability. Arguing that biological material and data should be considered a unified resource, these principles further enlarged in FAIR-Health,^9^ which includes (1) quality aspects related to research reproducibility and meaningful reuse of the data; (2) incentives to stimulate the active enrichment of data sets and biological material collections and reuse on all levels; and (3) privacy-respecting approaches when working with the human material and data.

In the context of (transnational) research, multiple legal and ethical questions routinely arise and require sound and yet practical solutions. Some of them are quite regular matters (e.g., seeking approval by the competent review ethics committee), whereas others are more challenging due to nature of the research question and/or due to the transnational dimension, therefore requiring special ethical and legal guidance. Questions may emerge during the proposal writing process, consortium agreement negotiations, or while addressing a specific research question. Whereas academic expertise in ethical and legal issues is quite comprehensive and publicly available, practical guidance, especially for transnational research, is often not (or not easily) to be found. We thus argue—after assessing available tools for ethical and legal guidance—that there is a need for a more practical, single-site knowledge base that promotes know-how through typical scenarios from research practice, ultimately to provide guidance for responsible research.

## Identifying and Assessing Tools for Ethical and Legal Guidance

In June 2014, different online tools and services intended to aid sample/data providers in navigating legal and ethical requirements related to the sharing of sensitive data and samples were discussed during a 1-day workshop in Berlin. It brought together and included the the BioMedBridges *Legal Assessment Tool* (LAT),^10^ the Biobanking and Biomolecular Resources Research Infrastructure (BBMRI) *legal WIK*,^11^ the *human Sample Exchange Regulation Navigator* (hSERN),^12^ and the P3G *International Policy interoperability and data Access Clearinghouse* (IPAC).^13^ Briefly, the discussion in this workshop showed “that mature tools to support sharing of sensitive samples and data are still lacking.”^14^ It is neither useful to simply quote articles of the law and leave it to the users, typically nonlegal experts, to make sense of them for their research, nor is it sufficient to provide a first orientation through a decision tree or tool, when researchers need specific answers for their project. Instead, the targeted users have to be well defined before appropriate ways to present the information to them can be developed.

In November 2015, developers and contributing experts of the three legal tools, WIKI, LAT, and hSERN, met to assess the main challenges encountered while conceptualizing, initiating, and maintaining the respective tools. Some key challenges are mentioned here: (1) Each of the tools came into being in the context of a particular research project and faced difficulties with regard to sustainability after the project ended. This could be on the level of IT support or expertise accuracy or both. (2) All of the tools shared the feature of not having narrowed down the potential users (e.g., researchers or legal experts) and lacked a certain user friendliness. (3) It was concluded that tools could only provide initial guidance and generic information, but that the need for customized advice from ethical, legal, and societal experts on a case-by-case basis should not be ignored. Based on the experience in developing, using, and assessing existing ethical, legal, and societal issue (ELSI) guidance tools, it has therefore become apparent that a single, more user-friendly, and sustainable knowledge base is needed.

In a next step, the same group of experts decided to take an experimental approach in trying to combine the content of all tools into one in the context of the H2020 project ADOPT BBMRI-European Research Infrastructure Consortium (ERIC).^15^ Technically, this would have immediately solved the sustainability issue as BBMRI-ERIC is able to provide a durable platform for the tool. However, it became apparent that first an entire reconceptualization toward a single, user-friendly ELSI support approach is required. Especially one that puts the user needs at the core: What is the professional background of individuals seeking ethical and legal guidance? Whom do they turn to? What is missing for them?

In 2016, an opportunity to identify users and user perspectives occurred in the context of the H2020 project CORBEL,^16^ which brings together eleven biological and medical science research infrastructures (BMS RIs) that aim to create a platform for harmonized user access to biological and medical technologies, biological samples, and data services. Biological and medical research that addresses the grand challenges of health and aging spans a broad range of scientific disciplines and user communities. The BMS RIs play a facilitating role when interdisciplinary biomedical and translational research requires particular resources—such as biobank samples, imaging facilities, molecular screening centers, or animal models—from multiple research infrastructures. To provide guidance on ethical and legal challenges that might occur in this context, CORBEL conceptualizes providing appropriate guidance by building on the BBMRI-ERIC Common Service ELSI,^17^ which provides tools and expertise as well as knowledge and sharing of expertise regarding ethical, legal, and societal issues with a focus on servicing the biobanking community.

Therefore, the CORBEL project provided the ideal platform for the exploratory user survey: *Where do you get support with ELSI questions around data and biosamples?* Circulated through the CORBEL BMS RIs, the survey consisted of 24 questions that aimed to assess if the tools and platforms researchers use in relation to ethical, legal, and societal issues were deemed informative for their daily work and which questions are yet to be answered. Considering the distribution of the survey via mailing lists of CORBEL as well as all BMS RIs, the turnout comprising 36 replies was relatively low. Nonetheless, the responses showed some clear results: any tool must be easily accessible, and the content must be comprehensible to researchers, as well as helpful in regard to their specific needs; the advice given must be reliable, that is, checked with experts and maintained and updated on a regular basis. Moreover, the primary users are nonlegal experts and instead are researchers who typically seek practical advice in relation to a particular research project. In conclusion, the survey supported the initial idea to create a single, more user-friendly and sustainable tool tailored for a precise user group. Furthermore, it suggests building a resource that allows a multilevel, comprehensive user support for investigators for their daily research practice.

## Toward a Comprehensive Resource

Providing such guidance in the field of legal and ethical framework of research involving humans is a challenging undertaking. This is because research often raises ethical and legal issues in a variety of fields, such as fundamental rights (e.g., Freedom of Research, Privacy, Nondiscrimination), physicians' professional codes, data protection law, genetic testing law, ethics committees remit by law, private law (the role of contracts and templates, self-commitments, legal capacity of minors and disabled, surprise clauses and understandability rules, electronic authorization/signature), IP law (data “ownership,” patentability). Typically, biomedical researchers are not aware of all relevant legal issues. Hence, the relevance of these and other related fields can hardly be explained to biomedical researchers in a mere frontal, theoretical way (e.g., by listing relevant statutes or other legal sources, which are only understandable to those trained in reading the law). No researcher is trained to differentiate between relevant and irrelevant rules concerning a concrete case and how these apply regarding the legal constraints of a concrete project. Instead, it is up to the legal experts to provide guidance on what rules are applicable and how they are applied in detail. However, providing such a translation for researchers of how the law is applicable in practice to researchers is equally not a simple exercise.

### Building on scenarios to show how law and ethics are applied

To be ultimately useful, the legal requirements must be examined and presented by legal experts, consistent with typical research situations that investigators can relate to. We thus propose that several real-life cases can be taken and transformed into an informative, archetypical situation (scenario). Such scenarios might be helpful to the researcher in various ways such as identifying and taking into account in an appropriate manner the actual ethical and legal challenges, ideally before data and samples are collected. Practice of ethical and legal guidance has shown that a lot of difficult situations could be avoided, if the rules that apply for sharing and accessing data from various sources would have been taken into account at the right moment, namely when conceptualizing the research projects. Whereas the envisaged benefits of data sharing are usually well described from a scientific perspective, the ELSI aspects are typically neglected, especially from a practical data sharing viewpoint.

The scenario-based approach can help to avoid such predicaments in the first place by raising awareness and providing guidance for the assessment upfront. The legal guidance can thus be provided by answering typical questions that arise for researchers when setting up a project as well as when trying to share data. The researcher will ideally find the relevant questions for his or her case by identifying the scenario, which is similar to his or her project or scientific undertaking. Through the relevant scenario, he or she will then come across the relevant issues that he or she might even not have been aware of before and will find guiding advice how to address them.

In the law, there will always be situations for which insufficient or nonconclusive answers are to be found. Consequently, our approach is based on the interrelationship of law and ethics. Practical information on how to comply with the legislation and ethical standards will be provided not just by referring to legal and ethical documents (e.g., statutes, guidelines and best practices) but also by integrating the ethical aspects into the presentation of archetypical scenarios that demonstrate which documents are relevant for which case and how these documents are best applied in practice. By doing so, it can also be shown how law and ethics interrelate and are complementary to each other.

Finally, the same scenario-based approach could be used to help researchers identify relevant procedures and authorities, as well as tools and mechanisms to connect legal orders when conducting cross-border research. The scenarios, for example, will also show in which context what authority (e.g., a local ethics committee) has to be involved and at what stage.

### Avoiding legalistic language to make the law accessible

Abstract terms and concepts, often seen as “legalistic” language, can be difficult to grasp and merely quoting relevant articles may prevent users from extracting and applying the relevant information accordingly. To make legal and ethical advice accessible, the law should be contextualized and explained in an accessible language. Consider, for instance, the term “data controller” defined as “natural or legal person, which determines the purposes and means of the processing of personal data” (Art. 4 (7) GDPR). Consequently, when exchanging data, the question that quite naturally arises for any principal investigator is if she or he is the data controller. Scenarios can help here as they use simplified language by introducing an archetypical story line with fictional characters and names and ultimately answer the question(s) raised. Consider the following example:

Scenario 1:

Lise is a medical doctor working for a university hospital specialized in cancer treatment based on novel approaches developed in the research department, which she is also a member of. The samples and data necessary for the research which she wants to conduct are stored in the local biobank. Lise is going to be the principal investigator. She will collaborate with three other colleagues based in three different institutions/countries in Europe and the US, who need access to the data and samples.

Question 1:

Who is the “data controller”?

Answer 1:

The “data controller” in this scenario is the university hospital Lise is working for. Whether she is the princpal investigator or not does not determine data controllership in the sense of the GDPR. All roles and obligations of the research partners will be specified in a contract (e.g., data transfer agreement).

A scenario-based story line may aim to clarify the different roles of the parties involved and specify their respective responsibilities. It may also help to understand and identify conflicting interests of the parties involved (e.g., researchers collecting the data and/or researchers analyzing the data, IT staff setting up a secure database, IT staff responsible for combining data, patients entrusting their data for research). Typically, a scenario will raise further, typically interconnected questions that are an intrinsic part of any transnational research and data exchange. In showcasing the complexity and what to do about it can smoothen the process for researchers in practice.

### Addressing frequently asked questions and promoting templates, how-to guides, best-practice-models, and scripts

Scenarios, however, are only one entry point to appropriate and useful information when providing guidance for ethical and legal challenges in daily research practice. Scenarios provide information on what the issues are. Frequently asked questions (FAQs), templates, and guidelines provide instruments in how to address them.

Allowing fast reading, FAQs clarify in a few paragraphs key issues to multiple user groups. Some users might be unfamiliar with a certain topic altogether and seek a good overview. Others might have some knowledge derived from participating in research projects, being a member of an ethics committee, or received training on the subject matter. They may want to remain up to date or learn more. A typical question (and related to the above scenario) is: Does the EU General Data Protection also apply if the data are transferred to the United States? Which safeguards have to be in place?^18^

A broad range of issues are also regulated by a contract. Some organizations, universities, or research facilities have drafted Mutual Transfer Agreements and Data Transfer Agreements and promote them in-house for their researchers to use in collaborative projects. Others are therefore grateful to rely on templates developed and applied by research projects such as BBMRI-LPC^19^ and RD-Connect^20^ or national working groups such as in Germany.^21^ Thus, a collection of well-curated templates and also how-to guides, best-practice models, and scripts (e.g., for societal engagement) shall be set up and promoted. An overview over the existing templates and whether and by whom they are approved, as well as experiences with similar scenarios, would provide major support for researchers as well as for ethics review committees.

## Conclusion

**FIG. 1.**
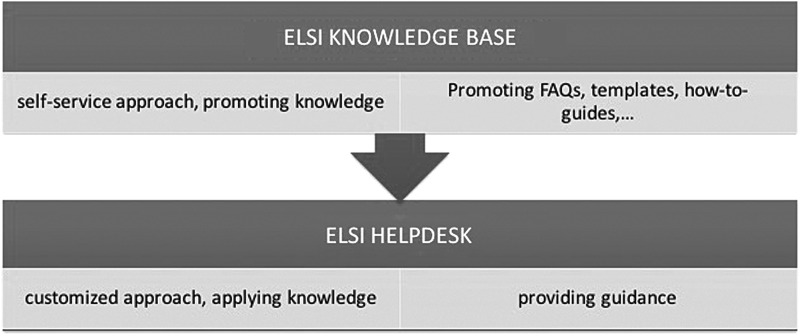
Relationship between Knowledge Base and Helpdesk.

Based on the experiences and assessments of ELSI guidance tools, which are excellent in their own right but limited in their usage, we proposed a reconceptualization that defines a clear user group and established the *ELSI Knowledge Base*. The ELSI Knowledge Base will be fully operational in Q4 2018. Generally speaking, a knowledge base is a store of information or data available to the user on a self-serve basis. The information in a knowledge base comes from multiple sources, usually from several expert contributors who are well versed on the subject matter. The knowledge base provides users with a first orientation on the subject matter, as well as allowing them to explore more detailed information if desired in a self-service manner. It is crucial that the information and knowledge provided are shared in a manner that is user friendly. Long lists of links, legalistic language, and multiple clicks have to be avoided wherever possible. The long-term sustainability and accuracy of a knowledge base need to be ensured by placing its expert curation and technical maintenance under the responsibility of an organization rather than a research consortium. In its core, it builds on a scenario-based approach using a nonlegalistic language. In addition, the knowledge base connects to FAQs, promotes contract and informed consent templates, how-to-guides, best-practice models, and scripts. The ELSI Knowledge Base is a key element of the BBMRI-ERIC Common Service ELSI, which currently serves biobanks but shall be enlarged to serve the BMS community. In contrast to the ELSI Helpdesk (Fig. 1), which provides customized support, the ELSI Knowledge Base is available to the user on a self-serve basis and expected to be fully operable in autumn 2018.

## Notes

^*^ The reasons for the delay can be manifold. Typical issues are the underestimated negotiation time for agreeing on a Mutual Transfer Agreement or different procedures and competencies for ethical approvals.
